# Engineering proteinaceous colloidosomes as enzyme carriers for efficient and recyclable Pickering interfacial biocatalysis†

**DOI:** 10.1039/d1sc03693a

**Published:** 2021-08-17

**Authors:** Hang Jiang, Xiaofeng Hu, Yunxing Li, Cheng Yang, To Ngai

**Affiliations:** The Key Laboratory of Synthetic and Biological Colloids, Ministry of Education, School of Chemical and Material Engineering, Jiangnan University Wuxi 214122 P. R. China yunxingli@jiangnan.edu.cn; Department of Chemistry, The Chinese University of Hong Kong Shatin, N. T. Hong Kong P. R. China tongai@cuhk.edu.hk

## Abstract

Despite Pickering interfacial biocatalysis being a popular topic in biphasic biocatalysis, the development of water-in-oil (w/o) emulsion systems stabilized by single particles remains a challenge. For the first time, hydrophobized proteinaceous colloidosomes with magnetic-responsiveness are developed to function as both an enzyme carrier and emulsifier, achieving a breakthrough in protein-based w/o Pickering bioconversion. Enzyme-loaded protein colloidosomes are synthesized by a facile and mild method *via* emulsion templating. This system exhibits superior catalytic activity to other systems at the oil–water interface. Besides, feasible enzyme recovery and reusability ensure that this novel system can be employed as an efficient and eco-friendly recyclable platform.

## Abstract 2

Engineering proteinaceous colloidosomes with magnetic-responsiveness are designed as both enzyme carrier and emulsifier, achieving a breakthrough in protein-based w/o Pickering interfacial biocatalysis.

## Graphical abstract

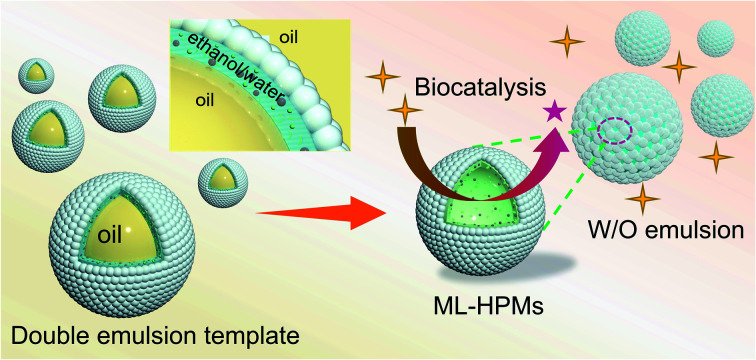

## Introduction

Enzymes have shown extraordinarily high catalytic activity and selectivity in chemical processes under mild and sustainable conditions.^1–5^ Because substrates in many situations are soluble in organic solvents and enzymes as a biocatalyst are often active in water, enzymatic reactions typically occur at the organic–aqueous biphasic interface, in which the interfacial area is the critical factor for the catalytic efficacy.^6,7^ One common and efficient method for increasing the oil–water contact area is to create emulsions, which are usually stabilized by surfactants.^8,9^ However, this technique is impeded by surfactant-induced inhibition of enzyme activity and difficulty with product separation.^10^

Particle-stabilized emulsions, also known as the Pickering emulsion, have received considerable interest in biphasic enzymatic catalysis.^11–29^ The novel platform combines the advantages of enhanced stability, improved biocompatibility, and ease of product/catalyst separation.^13,14^ Given that the involved reactants are organic-soluble, water-in-oil (w/o) Pickering emulsions with oil as the continuous phase are more preferable for feasible extraction of products and reuse of catalysts. In pioneering work, Wu *et al.* utilized silica nanoparticles as emulsifiers to prepare a w/o Pickering emulsion with an enzyme loaded in the internal aqueous phase for biocatalysis.^20^ Further, van Hest *et al.* reported versatile w/o systems stabilized by polymersomes and demonstrated the difference brought about by the enzyme contained in the lumen as the enzyme was simultaneously brought to the w/o interface during emulsification. As a result, the specific activity of the enzyme was demonstrated to be significantly higher than that of a common biphasic system when the enzyme was encapsulated in the aqueous phase,^21^ which is the rudiment of Pickering interfacial biocatalysis (PIB). Recently, PIB has gained popularity and efficiency in biphasic biocatalysis due to its advantages of (1) increased enzyme utilization, (2) shortened mass transfer, and (3) facilitated enzyme recycling.^22–29^

In essence, enzymatic colloidal particles are the central building block of a successful PIB system (a particulate stabilizer loaded with enzyme). As a type of active protein, enzymes are usually soluble in aqueous liquids.^30^ On this account, most enzyme immobilization processes should take place in an aqueous environment, which necessarily requires particles to be well dispersed in water. Therefore, hydrophilic particles are the best choice. For instance, enzymes can be brought to the oil–water interface *via* immobilizing in porous carbon/silica nanoparticles,^23,24^ entrapment in capsules and colloidosome,^25,26^ conjugation with metal–organic frameworks (MOFs) or polymers.^3,27,28^ However, enzyme-loaded hydrophilic particles are specifically limited with respect to the formation of oil-in-water (o/w) emulsions. Recently, we synthesized poly(2-(diethylamino)ethyl methacrylate) (PDEAEMA) microgels to enclose enzymes for biphasic catalysis.^29^ Due to the low stability of w/o emulsions exclusively stabilized by microgels, hydrophobic silica nanoparticles were used as a co-stabilizer to improve the emulsion stabilization. Thus, the development of sole particles for the preparation of a stable w/o PIB system in a facile and mild manner remains a great challenge.

Zein is a green and nontoxic plant protein, with outstanding biocompatibility and sustainability.^31^ In particular, zein-based materials are promising candidates in drug delivery, bio-actives encapsulation, biodegradable materials, *etc.*^32–35^ Due to its special solubility, zein can dissolve/precipitate along with the change of the solvent composition, and the protein molecules can precipitate into proteinaceous colloidal particles for preparation of o/w Pickering emulsions.^36–39^

Herein, hydrophobized protein microspheres were designed for the first time to act as both a colloidal emulsifier and enzyme carrier, achieving a breakthrough in protein-based w/o PIB systems. A proteinaceous colloidosome structure was formed using an oil-in-(ethanol/water)-in-oil double emulsion stabilized by commercially-available hydrophobic silica nanoparticles with zein as the skeleton, physically and simultaneously modifying the colloidal proteinaceous stabilizer with hydrophobicity and enzyme immobilization. Furthermore, magnetic responsiveness was incorporated into the design of the colloidal particle for quick product-catalyst separation. The use of magnetic carriers for enzymes, particularly for biocatalysis, is regarded as a desirable procedure that is both convenient and environmentally acceptable.^40–42^ It avoids other stimuli-responsive actions such as pH regulation and temperature change, which may destabilize the emulsion or deactivate enzymes.^14,43^ The enzymatic activity can be preserved to a significant extent due to physical trapping by the emulsion template; furthermore, hydrophobic silica nanoparticles effectively alter the surface properties of protein microspheres for successful stabilization of w/o emulsions. As a result, proteinaceous colloidosomes were engineered with characteristics of emulsifying ability, enzymatic activity, and magnetic responsiveness in a green and facile manner.

## Results and discussion

**Scheme 1:**
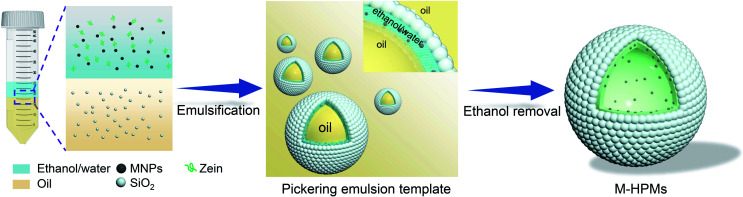
Schematic illustration of preparation of magnetically hydrophobized protein microspheres with a colloidosome structure (M-HPMs) from Pickering double emulsions.

The process for the fabrication of magnetically hydrophobized protein-colloidosome microspheres (M-HPMs) is schematically illustrated in Scheme 1. First, magnetic nanoparticles (MNPs) were dispersed in zein–ethanol–water solution used as magnetic-responsive sites in M-HPMs. Following that, the ethanol/water phase was mixed with the oil phase containing hydrophobic silica nanoparticles for emulsification. A Pickering double emulsion template was then successfully fabricated, in which the ethanol/water phase acted as the middle phase. Eventually, M-HPMs were successfully prepared by removal of ethanol and interior oil, avoiding chemical polymerization or protein crosslinking. During this process, with the composition change of the ethanol/water phase, zein molecule precipitates for shaping the skeleton-structure to ensure intact protein-colloidosome microspheres. Despite a stable emulsion template being generated using only hydrophobic silica nanoparticles (Fig. S1†), the microspheres cannot be obtained in the absence of zein. As for the hydrophobic silica nanoparticles, a crucial part for protein-colloidosome microspheres, which were not only employed as a solid emulsifier, but also played a significant role in effectively tailoring the wettability of protein microspheres by anchoring on the surface. In accordance with our previous finding,^44^ a double emulsion was produced using the hydrophobic emulsifier and zein. Differently, the emulsion template in this work gained advantages from the irreversible adsorption mechanism of particles. After storage for one month, the droplets retained excellent stability (Fig. S2†), which was conducive in preventing droplets from coalescence during ethanol removal.

**Fig. 1:**
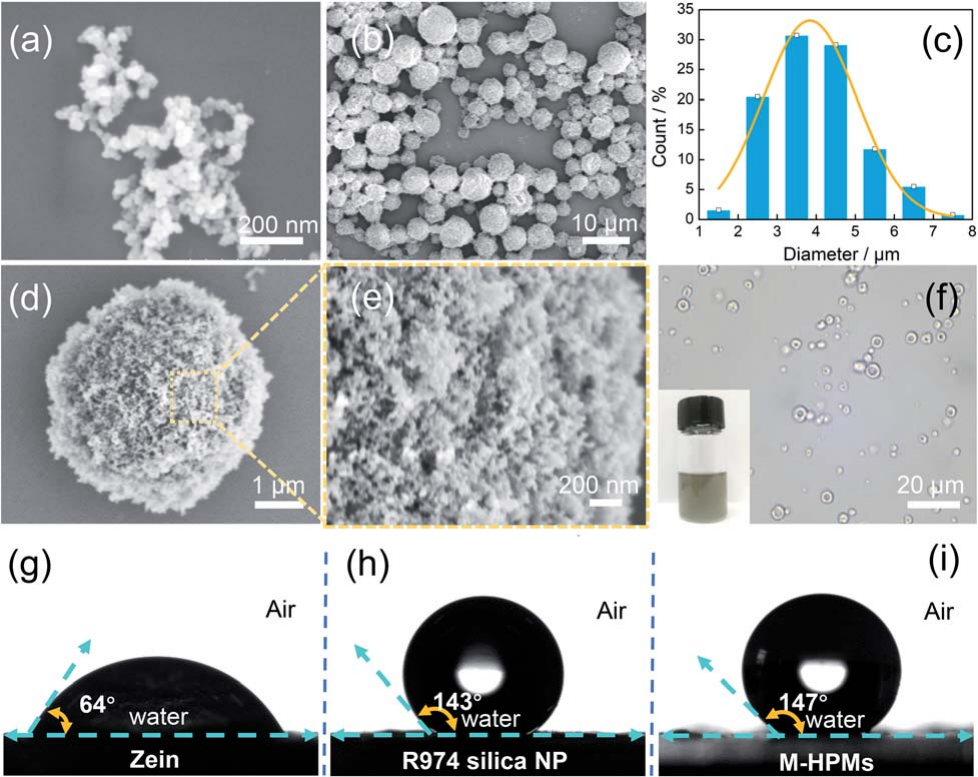
(a) SEM image of hydrophobic silica nanoparticles, (b) SEM image and (c) size distribution of M-HPMs, (d) and (e) SEM images of M-HPMs under high magnification, and (f) optical microscope image of M-HPMs. Air–water contact angles of (g) zein powder, (h) hydrophobic silica nanoparticles (R974) and (i) M-HPMs.

According to scanning electron microscopy (SEM) images, M-HPMs produced with 1% hydrophobic silica nanoparticles showed a relatively uniform size of approximately 3.9 ± 1.18 μm on an average (Fig. 1b and c). At high magnification, the rough surface of M-HPMs was clearly visible in Fig. 1d and e, where an array of hydrophobic silica nanoparticles (Fig. 1a) densely covered the whole zein scaffold. Because of the hydrophobized surface of the colloidosome microspheres, the M-HPMs dispersed satisfactorily in toluene (inset), as seen in Fig. 1f. More importantly, when compared to natural zein protein, hydrophobic silica nanoparticles imparted a large contact angle to M-HPMs (>140°), as displayed in Fig. 1g–i, enabling stabilization of w/o Pickering emulsions with enough hydrophobicity. Owing to the co-precipitation with MNPs, the M-HPMs also exhibited a rapid magnetic response (Fig. S3†). Besides, it was found that the size of the M-HPMs was appropriately controlled by the varied amount of hydrophobic silica nanoparticles (Fig. S4†).

**Fig. 2:**
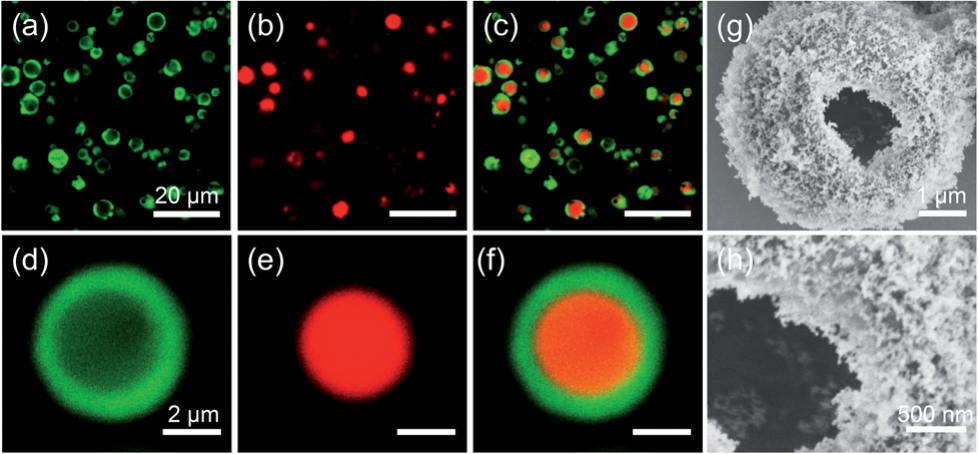
(a)–(f) CLSM images of M-HPMs at different magnifications: (a) and (d) the zein was labelled by FITC under a green channel, (b) and (e) the oil was stained by Nile red under a red channel and (c) and (f) the overlays of two stained images. (g) and (h) SEM images of broken M-HPMs. Scale bars are (a)–(c) 20 μm, (d)–(f) 2 μm.

We stained the zein solution with fluorescein isothiocyanate (FITC) and the oil phase with Nile Red to visualize the interior structure more directly. Following emulsification and evaporation of ethanol, the dispersion was diluted with fresh oil and observed using confocal laser scanning microscopy (CLSM). As illustrated in Fig. 2a–c, the majority of obtained microspheres contained a single red oil droplet inside, surrounded by a characteristic green circle representing a thick shell of the precipitated zein protein. Apart from this, a hollow architecture can be clearly observed from a broken microsphere, as seen in Fig. 2g and h, where a thick zein shell was completely visible through the crack. Clearly, the development of the zein shell resulting from the double emulsion template not only strengthens the structural stability of the microsphere, but also allows for the hydrophobic silica nanoparticles to anchor on it, forming a colloidosome structure. We speculate that the formation of multiple emulsions is attributed to the presence of dual emulsifiers, and zein protein helps to stabilize the oil-in-(ethanol/water) emulsion, while the hydrophobic silica nanoparticle helps to stabilize the (ethanol/water)-in-oil emulsion.

**Fig. 3:**
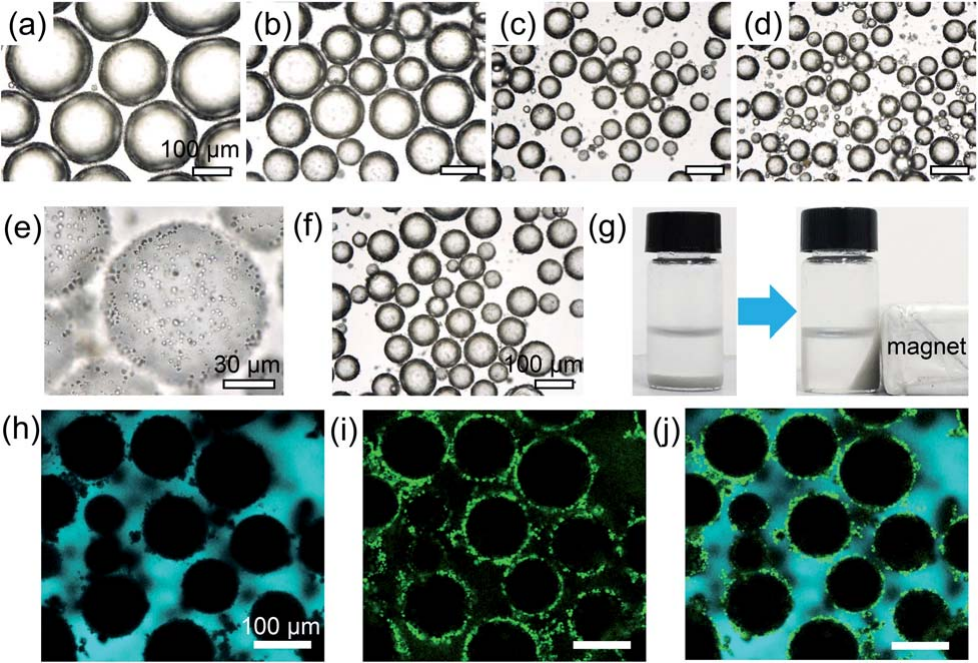
Optical microscope images of Pickering emulsions stabilized by ML-HPMs at different concentrations, respectively: (a) 0.25%, (b) 0.5%, (c) 1%, and (d) 2%; the ratio of toluene to water was 3 : 1. All scale bars are 100 μm; (e)–(j) further characterization of (c): optical microscope images of the (e) interfacial structure of droplets and the (f) emulsion after one month storage, (g) exhibition of magnetic responsiveness, and (h)–(j) CLSM images; ML-HPMs and toluene were stained with FITC (green) and pyrene (cyan), respectively; all scale bars are 100 μm.

**Fig. 4:**
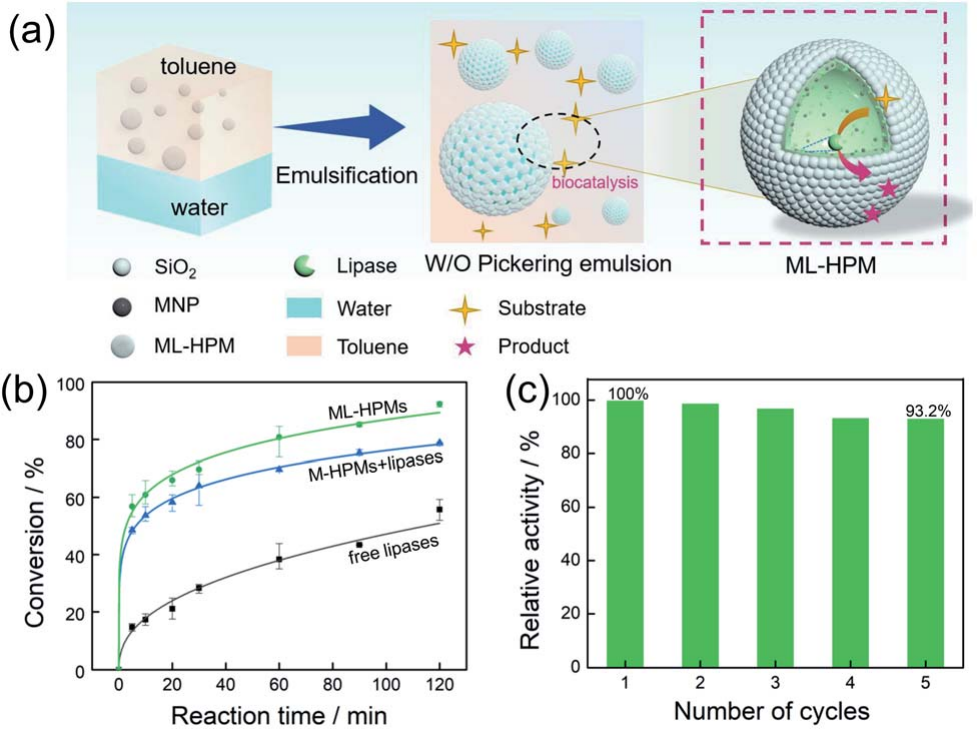
(a) Schematic illustration of interfacial catalysis in the water-in-toluene Pickering emulsions stabilized by 1% ML-HPMs. (b) Conversion of 1-hexanol and hexanoic acid to ester with the reaction time for free lipase in a water–toluene biphasic system; free lipase was in the water phase of the w/o Pickering emulsion stabilized by M-HPMs and the w/o Pickering emulsion stabilized by ML-HPMs. (c) Reusability of ML-HPMs for 5 cycles.

As the interfacial area is critical for the catalytic process of PIB, it was necessary to assess parameters affecting emulsion stabilization and the morphology. We initially investigated the oil/water volume ratio and observed a declining trend in the size of emulsion droplets as the oil fraction increased, but excess particles were observed in the exterior phase at high oil/water ratios (>3 : 1); meanwhile the difference in droplet diameter was small, indicating that there were sufficient microspheres for emulsion stabilization (Fig. S5 and S6†). Therefore, a 3 : 1 ratio was chosen for the following investigation. As illustrated in Fig. 3a–d, lipase was carried in the M-HPMs for the stabilization of w/o Pickering emulsions at various concentrations, and the proteinaceous colloidosomes exhibiting both magnetic and enzymatic activity was denoted as ML-HPMs. By adding 0.25% (w/v) ML-HPMs, the mean diameter of the emulsion droplets was measured to be 203 ± 24.6 μm (Fig. 3a), while increasing the amount of ML-HPMs (1%) significantly reduced the size of the droplets to 74 ± 16.5 μm (Fig. 3c and 4b). As demonstrated in Fig. 3d, further increasing the colloidosome concentration had little influence on the size and morphology of the resulting emulsion. Additionally, the redundant ML-HPMs tended to aggregate in the continuous phase. Therefore, an appropriate concentration (1%) with a 3 : 1 oil/water ratio was readily chosen for further study.

The magnified microscopy image clearly depicts the interfacial structure of the emulsion droplet (Fig. 3e). A considerable amount of ML-HPMs compactly covered the aqueous droplet, simultaneously serving as a stabilizer and biocatalyst. As illustrated in Fig. 3f, the mean diameter of droplets remained nearly unchanged after one month of storage, suggesting outstanding resistance to coalescence. Additionally, due to the trapped magnetic particles within the proteinaceous colloidosomes, the resultant w/o emulsion exhibited rapid magnetic collection, as demonstrated in Fig. 3g. During the preparation process, the oil phase was stained with fluorescent pyrene and the proteinaceous colloidosomes were labelled with FITC. As seen in Fig. 3h, the exterior cyan colour indicated that the emulsion was w/o type. Additionally, green fluorescent dots circling the w/o interface demonstrated the excellent adsorption activity of ML-HPMs (Fig. 3i and j), creating a desirable biphasic platform for enzymatic catalysis.

To evaluate the biocatalytic behaviours of the PIB system, a representative esterification reaction between 1-hexanol and hexanoic acid was selected as a model. The overall process of interfacial biocatalysis is depicted in Fig. 4a, and the whole process of bioconversion occurred exclusively at the w/o interface without the need for a long distance of mass transfer. Two classical control systems were set up for comparison. Among them, identical reactions were carried out with the same quantity of substrates. One was a normal biphasic system without emulsification, and the other was an M-HPMs stabilized Pickering system in which the enzyme was encapsulated inside water droplets, with both systems containing an equal amount of enzyme in the aqueous phase. As shown in Fig. 4b, the conversion efficiency in three systems progressively increased with reaction time. Particularly, for the two Pickering systems, in the first 5 min the conversion increased to 48.6% and 56.8%, but there was only 14.6% in the biphasic system. Hence, the catalytic performance remarkably benefited from the large interfacial area generated by the emulsions. In particular, the PIB system exhibited superior interfacial catalytic performance, achieving 92.4% after 120 min. In comparison, the conversion in the Pickering emulsion with conventional enzyme encapsulation inside only reached 78.9%.

Enzyme reusability is another important issue regarding PIB systems, but catalyst circulation is generally difficult and time-consuming.^45^ For example, purification is a common problem in surfactant-stabilized systems; in addition, high-energy centrifugation is a most frequently employed procedure for product separation and enzyme recovery, but is tedious since pre-balancing is required, and may demulsify some fragile emulsions (needing secondary emulsification).^46^ Due to the incorporated magnetic responsiveness of the particulate stabilizer, the w/o emulsion here can be fast collected by magnetic adsorption and separated for next catalytic cycles. In such a rapid and mild process, the emulsion together with the enzyme was reused over five consecutive cycles and the relative catalytic conversion remained high (Fig. 4c, from 100% to 93.2%), indicating that efficient recovery and desirable reusability can be accomplished. We supposed that the slight decrease of catalytic activity is probably attributed to enzyme denaturation. Moreover, cascade catalysis by multi-enzymes is highly anticipated for future research, as the inner aqueous phase can potentially be utilized for encapsulation of another enzyme.

## Conclusions

In summary, a novel w/o PIB system solely stabilized by engineering proteinaceous colloidosomes was first proposed in this work. By introducing hydrophobic silica nanoparticles on the surface of the colloidosomes *via* emulsification, the challenge of stabilizing w/o emulsions using sole particles was completely addressed. Meanwhile, magnetic nanoparticles and lipase were simultaneously incorporated into the proteinaceous colloidosomes, imparting interfacial catalysis and magnetic response to the w/o emulsion. In comparison to both conventional biphasic systems and w/o Pickering emulsions with enzyme located inside, the PIB system demonstrated: (1) enhanced catalytic activity; (2) ease of product separation; and (3) exceptional recyclability. As the w/o emulsion template for formation of such proteinaceous colloidosomes can be easily realized by diverse particles, we envisage that this novel concept will be utilized as a versatile technique for the synthesis of eco-friendly and renewable materials for a variety of uses.

## Data availability

Experimental data associated with this article have been provided in the ESI.†

## Author contributions

H. Jiang: conceptualization, investigation, methodology, supervision, and writing – review & editing; X. Hu: data curation, investigation, resources, and writing – original draft; Y. Li: conceptualization, project administration, writing – review & editing, and supervision; C. Yang: writing – review & editing; T. Ngai: writing – review & editing, supervision, and funding acquisition.

## Conflicts of interest

There are no conflicts to declare.

## Supplementary Material

SC-012-D1SC03693A-s001
