# Supplementary material for: Engineering proteinaceous colloidosomes as enzyme carriers for efficient and recyclable Pickering interfacial biocatalysis
Source: Chem Sci. 2021 Aug 17;12(37):12463–7. doi: 10.1039/d1sc03693a (PMC8480340; doi:10.1039/d1sc03693a)
Supplement: SC-012-D1SC03693A-s001 [file SC-012-D1SC03693A-s001.pdf]

Electronic Supplementary Material (ESI) for Chemical Science.
This journal is © The Royal Society of Chemistry 2021

Engineering proteinaceous colloidosomes as enzyme carrier for efficient and recyclable Pickering interfacial biocatalysis

Hang Jiang,[†a] Xiaofeng Hu,[†a] Yunxing Li,[*a] Cheng Yang,[a] and To Ngai[*a,b]

[a] *The Key Laboratory of Synthetic and Biological Colloids, Ministry of Education & School of Chemical and Material Engineering, Jiangnan University, Wuxi 214122, P. R. China.*

[b] *Department of Chemistry, The Chinese University of Hong Kong, Shatin, N. T., Hong Kong, P.R. China.*

† These authors contributed equally to this work.

* E-mail: yunxingli@jiangnan.edu.cn

* E-mail: tongai@cuhk.edu.hk

1. Materials

Zein (grade Z3625), Nile Red and lipase from *Candida sp.* expressed in *Aspergillus niger* were purchased from Sigma-Aldrich (USA). R974 silica nanoparticles were purchased from Evonik Industry Co., Ltd (Germany). Hexanoic acid (>99%) and 1-hexanol (>99%) were bought from Tokyo Chemical Industry Co., Ltd (Japan). Fluorescein isothiocyanate (FITC) and Fe_3O_4 nanoparticles (magnetic nanoparticles, MNPs) were acquired from Macklin Biochemical Co., Ltd (China). Pyrene was gotten from Aladdin Chemical Reagent Company (China). A mixture of caprylic/capric triglycerides (GTCC) was obtained from Guangzhou Chou Qin Biotechnology Co., Ltd

(China). Ethanol (AR, >99.7%), n-hexane, n-octanol, toluene, sodium dihydrogen phosphate (99%) and sodium phosphate dibasic (99%) were supplied by Sinopharm Chemical Reagent Co., Ltd (China). All chemicals were used as received. Deionized water (Milli-Q grade, 18.2 MΩ•cm) was used in all experiments.

2. Preparation of magnetically proteinaceous microspheres with colloidosome structure (M-HPMs).

M-HPMs were prepared via a similar method as our previous work. First, a certain amount of zein powder was dissolved in ethanol-water solution (70 vol%) with ultrasonic to prepare a 20% (w/v) zein stock solution. 0.5% (w/v) magnetic nanoparticles (MNPs) were dispersed in zein solution to obtain ethanol/water phase. The oil phase was prepared by mixing 0.2 g of silica nanoparticles with 20 mL of GTCC. Pre-emulsification was performed by adding 4 mL of the zein stock solution to 20 mL of the oil phase under vortex stirring for 1 min. The resulting mixture was then emulsified by homogenization under 18000 rpm for 2 min. Subsequently, an as-prepared double emulsion was rapidly transferred to a flask. The ethanol was removed under reduced-pressure rotary evaporation, during which hollow microspheres of zein gradually precipitated. The microspheres were washed several times with n-hexane to remove interior oil. Finally, the microspheres were collected by vacuum dried at 50°C for 12 h. FITC and Nile Red (0.1 mg/mL, based on the corresponding phase volume) were added to the aqueous phase and oil phase, respectively, to enable confocal microscopic characterization of these emulsions and particles.

3. Contact Angle Measurement

The sessile drop method was used to measure the air-water contact angle of zein and the particles. In brief, the zein powder was compressed into flakes as sample, for silica nanoparticles and M-HPMs, the particles were dispersed in n-hexane to form 3% (w/v)

dispersion, respectively. The dispersion was dropwise injected onto the clean coverslip and naturally dried, to obtain a uniform sample coating. Then, 2 μL of water droplet was deposited on each sample coating surfaces for measuring the contact angles. All samples were measured three times.

4. Preparation of magnetic proteinaceous colloidosomes loaded with lipase (ML-HPMs).

According to the above-described procedures, firstly, adjust the zein stock solution to pH 7.0, then 0.5%wt MNPs was adding into zein stock solution with ultrasonic, 800 μL of lipases solution were diluted and subsequently added into the mixture. Afterwards, following the steps-above homogeneous emulsification and rotary evaporation were performing. ML-HPMs were obtained after washing by n-hexane and freeze-drying.

5. Preparation of w/o Pickering Emulsion Stabilized by ML-HPMs.

Briefly, 1.0 mL phosphate buffer (pH 7.3) was adding into 3.0 mL ML-HPMs toluene dispersion at certain concentration. The mixture was then emulsified by vortex stirring for 2 min, forming a Pickering emulsion. Pyrene (0.1 mg/mL) was added into toluene for fluorescent view.

6. Assessment of Catalytic Performance.

The catalytic performance of the PIB system was evaluated by the esterification of 1-hexanol with hexanoic acid. In a typical experiment, 15.0 mL toluene solution containing 1-hexanol and hexanoic acid (0.1 M) was treated as oil phase and adding 5.0 mL phosphate buffer (pH 7.3), forming w/o Pickering emulsion stabilized by ML-HPMs (1% w/v, based on oil volume) as mentioned above. The control groups were conventional biphasic system consisting of lipase and substrate solution, and the Pickering emulsion stabilized by M-HPMs. In both experiments lipase was mixing in

phosphate buffer used as water phase. After each reaction cycle reacts for 2 h, the emulsion can be recovered by magnetic control and the oil phase was removal, and the emulsion was washing by toluene for at least three times, then the fresh substrate solution was added as oil phase for next reaction cycle. The concentrations of the substrates (1-hexanol, hexanoic acid) and product (hexyl hexanoate) were determined using a Shimadzu GC system (Nexis GC-2030) equipped with a flame ionization detector (FID).

7. Characterization

The morphology and size distribution of the colloidosomes were determined by scanning electron microscopy (SEM; Hitachi S-4800; Japan) and optical microscopy (VHX-1000C, Keyence; China). The concentrations of the substrates and products were determined by Nexis GC-2030 (Shimadzu, Japan) equipped with a flame ionization detector (FID) on a column (DB-FFAP column, 30.00 m length, 0.32 mm inner diameter, 0.50 μm film thickness). All fluorescence images were obtained by confocal laser scanning fluorescence microscopy (CLSM; TCS SP8; Leica; Germany), exciting the green fluorescent channel at 488 nm for FITC, red fluorescent channel at 530 nm for Nile red and blue fluorescent channel at 405 nm for pyrene. The size analysis of particles and emulsion droplets were calculated by image J software.

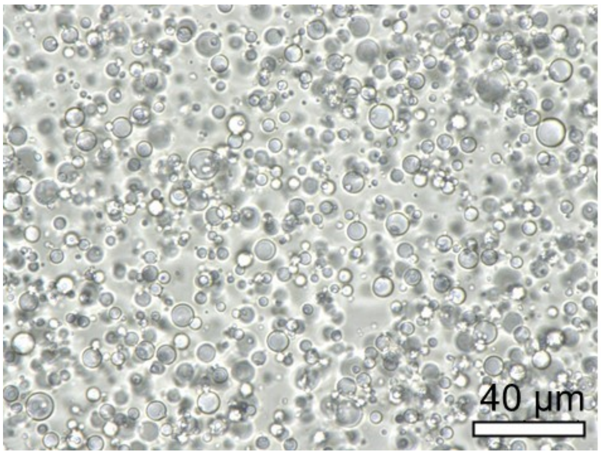

Figure S1. Optical microscope image of Pickering emulsion template solely stabilized by silica nanoparticles.

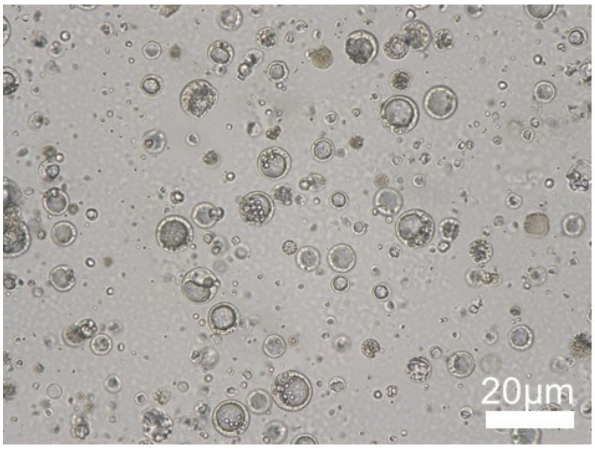

Figure S2. Optical microscope image of Pickering double emulsion template after one month storage.

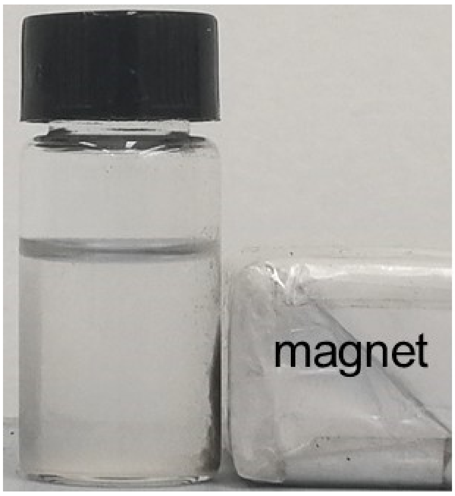

Figure S3. The magnetically responsive exhibition of M-HPMs, the microspheres were dispered in toluene.

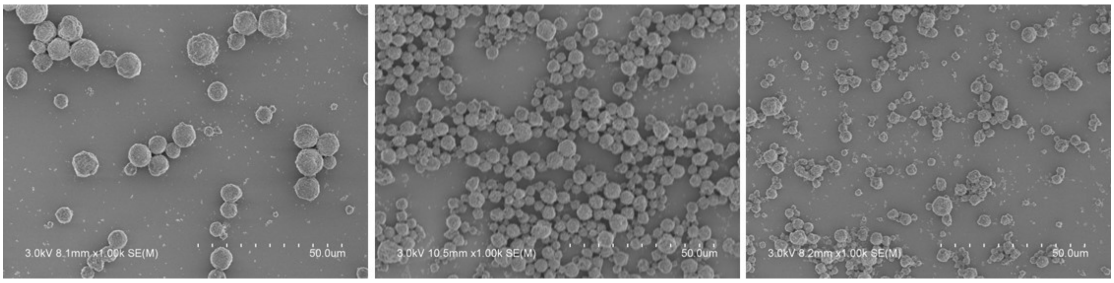

Figure S4. SEM images of M-HPMs prepared at different amounts of silica nanoparticles: (a) 0.5%, (b) 1% and (c) 2%.

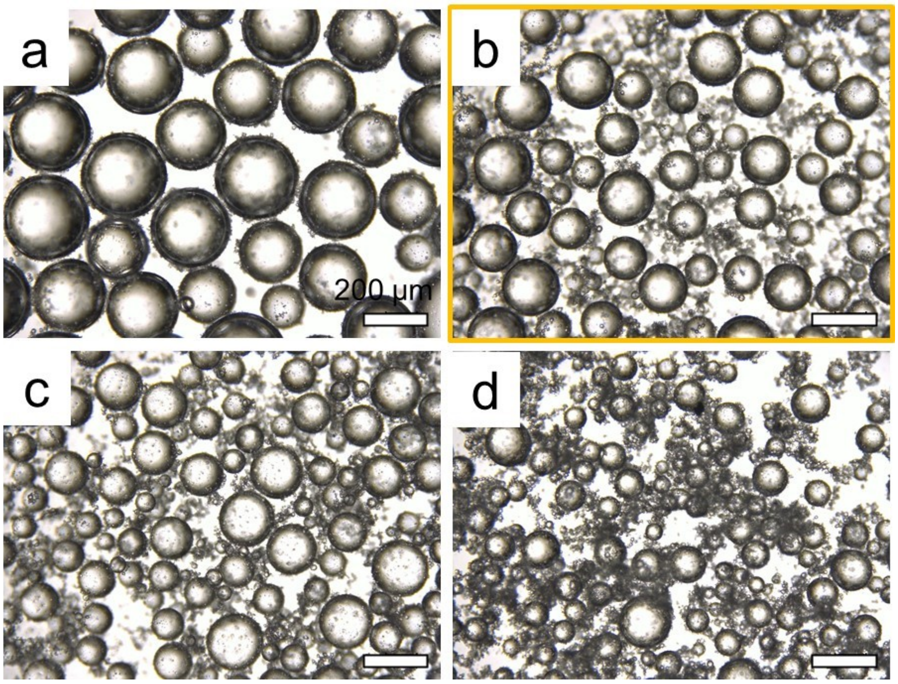

Figure S5. Optical images of water in toluene emulsions stabilized by ML-HPMs at different ratio of oil to water: (a) 1:1, (b) 3:1, (c) 5:1 (d)10:1.

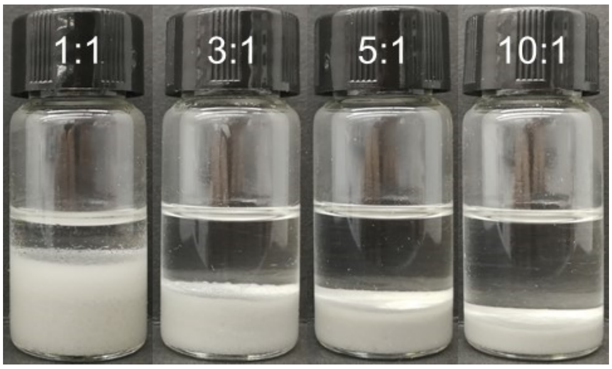

Figure S6. The appearance photos of water in toluene emulsions stabilized by ML-HPMs at different ratio of oil to water: (a) 1:1, (b) 3:1, (c) 5:1 (d)10:1.
